# Healthcare Resource Utilization and Associated Costs in Patients With Systemic Lupus Erythematosus Diagnosed With Lupus Nephritis

**DOI:** 10.7759/cureus.37839

**Published:** 2023-04-19

**Authors:** Christopher F Bell, Benjamin Wu, Shirley P Huang, Bernard Rubin, Carlyne M Averell, Benjamin Chastek, Erin M Hulbert, Joan Von Feldt

**Affiliations:** 1 US Value, Evidence and Outcomes, GSK, Durham, USA; 2 US Medical Affairs, GSK, Durham, USA; 3 Life Sciences, Health Economics and Outcomes Research (HEOR), Optum, Eden Prairie, USA; 4 US Medical Affairs, GSK, Philadelphia, USA; 5 Division of Rheumatology, Perelman School of Medicine at the University of Pennsylvania, Philadelphia, USA

**Keywords:** systemic lupus erythematosus, renal lupus, lupus nephritis, nephritis, healthcare resource utilization, healthcare costs

## Abstract

Background: Lupus nephritis (LN) is among the most severe organ manifestations of systemic lupus erythematosus (SLE), affecting between 31% and 48% of patients, usually within five years of SLE diagnosis. SLE without LN is associated with a high economic burden on the healthcare system, and although data are limited, several studies have shown that SLE with LN could increase this burden.

Aim: We aimed to compare the economic burden of LN versus SLE without LN among patients managed in routine clinical practices in the USA and describe the clinical course of these patients.

Materials and methods: This was a retrospective observational study of patients with commercial or Medicare Advantage health insurance. It included 2310 patients with LN and 2310 matched patients who had SLE without LN; each patient was followed for 12 months after diagnosis (the patient’s index date). Outcome measures included healthcare resource utilization (HCRU), direct healthcare costs, and SLE clinical manifestations.

Results: In all healthcare settings, the mean (SD) use of all-cause healthcare resources was significantly higher in the LN versus SLE without LN cohort, including the mean number of ambulatory visits (53.9 (55.1) vs 33.0 (26.0)), emergency room visits (2.9 (7.9) vs 1.6 (3.3)), inpatient stays (0.9 (1.5) vs 0.3 (0.8)), and pharmacy fills (65.0 (48.3) vs 51.2 (42.6)) (all p<0.001). Total all-cause costs per patient in the LN cohort were also significantly higher compared with the SLE without LN cohort ($50,975 (86,281) vs $26,262 (52,720), p<0.001), including costs for inpatient stays and outpatient visits. Clinically, a significantly higher proportion of patients with LN experienced moderate or severe SLE flares compared with the SLE without LN cohort (p<0.001), which may explain the difference in HCRU and healthcare costs.

Conclusion: All-cause HCRU and costs were higher for patients with LN than for matched patients with SLE without LN, highlighting the economic burden associated with LN.

## Introduction

Systemic lupus erythematosus (SLE) is a chronic, heterogeneous autoimmune disorder that predominantly affects women of reproductive age [1,2]. Diagnosis of SLE can be difficult: early symptoms are variable and not specific, the disease may involve several different organs, and often there are fluctuations between periods of active and quiescent disease [3].

Lupus nephritis (LN) is among the most severe organ manifestations of SLE, affecting between 31% and 48% of patients post-SLE diagnosis, usually within the first five years of diagnosis [4]. Additionally, up to 28% of patients with LN can progress to end-stage kidney disease (ESKD) [4]. The risk of death in patients with LN is six times that of the general population, increasing to up to 26 times in patients with ESKD [5]. Patients with SLE have a high burden of flares (episodes of increased disease activity), with ~95% of patients experiencing at least one flare in the first year post-diagnosis [6]. In patients with LN, the occurrence of renal flares is associated with progressive long-term kidney damage [7-9], and just a single renal flare can cause nephron loss and kidney damage that can impact the lifespan of the kidney [10].

SLE is associated with a high economic burden on the healthcare system, with mean annual all-cause healthcare costs per patient in the USA between US$17,258 and US$63,022 [11-14]. Although data are somewhat limited, several studies have shown that patients with LN incurred between two and four times the healthcare resource utilization (HCRU) and costs compared with patients with SLE without LN and between two and six times the costs of matched controls without SLE [15-18]. In two of these studies, the increase in annual healthcare costs for patients with LN versus patients with SLE without LN was determined primarily by inpatient admissions, pharmacy services, and outpatient care [15,18]. Similarly, a recent study reported that a quarter of patients with LN had ≥1 hospitalization in the 12 months following diagnosis, and the total mean all-cause healthcare costs per patient per year were $45,469 [19].

The aims of this study were to compare the economic burden of LN versus SLE without LN among patients managed in routine clinical practices in the USA and describe the clinical course of these patients. Some of the data reported in this paper have been presented as a poster at the American Society of Nephrology (ASN) 2020 (October 22-25, 2020), State of Texas Association of Rheumatologists (STAR) 2021 (February 26-28, 2021), Association of Women in Rheumatology (AWIR) 2021 (August 12-15, 2021), and Congress of Clinical Rheumatology-East and West (CCR-E and CCR-W) 2021 (CCR-E: August 12-15, 2021; CCR-W: September 18-21, 2021) annual meetings.

## Materials and methods

Study design

**Figure 1:**
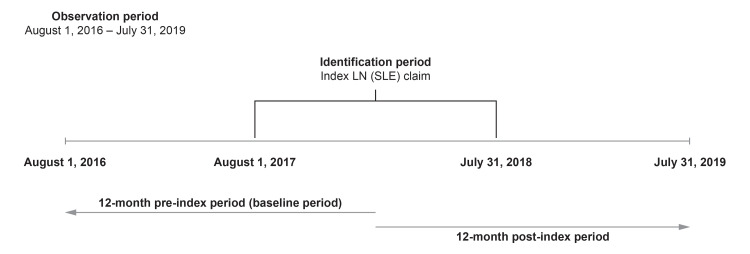
Study design. LN: lupus nephritis; SLE: systemic lupus erythematosus.

This was a retrospective observational cohort study (GSK study 213062) conducted between August 1, 2016, and July 31, 2019, using the Optum Research Database (Figure 1). This is a US-based claims database containing de-identified medical and pharmacy claims data for commercial and Medicare Advantage insurance enrollees, which prevents patient identification. This study complied with all applicable laws regarding patient privacy, and no direct patient contact or primary collection of individual patient data occurred. Due to this study's design, informed consent, ethics committee or institutional review board approval was not required.

Study population

Patients were assigned to the LN cohort or SLE without LN cohort between August 1, 2017, and July 31, 2018 (identification period), using specific International Classification of Diseases (ICD)-10 codes. LN was not biopsy-confirmed as claims databases such as the one employed in this study do not contain laboratory and procedure results (e.g., biopsy results). In lieu of biopsy results, we developed a definition based on a previously validated claims algorithm to identify patients with LN [20]. For the LN cohort and SLE without LN cohort, the index date was the date during the identification period of the first claim with a renal diagnosis code or an SLE diagnosis code, respectively. The LN cohort comprised patients who had ≥2 diagnoses of renal conditions on non-diagnostic claims during the identification period and had ≥1 diagnosis of SLE in an inpatient stay or ≥2 outpatient diagnoses of SLE that were ≥30 days apart in the 12-month pre-index date (baseline period). The SLE without LN cohort comprised patients who had no diagnosis of LN during the identification or baseline periods and had ≥1 inpatient stay or ≥2 medical claims with a diagnosis of SLE that was ≥30 days apart during the identification period.

All patients were ≥18 years of age as of the index year, had been continuously enrolled in a health plan with medical and pharmacy coverage for ≥12 months prior to the index as well as for the 12-month period after the index date, and had valid and non-missing demographic variables. Patients who were pregnant and/or had human immunodeficiency virus or acquired immunodeficiency syndrome (identified using the following codes: Healthcare Common Procedure Coding System, Current Procedural Terminology, revenue, and ICD-9 and ICD-10 procedure and diagnosis codes) during the identification period were excluded.

Outcomes

Baseline patient characteristics were assessed in the 12-month baseline period, and renal laboratory test results (proteinuria, protein/creatinine ratio (uPCR), and estimated glomerular filtration rate (eGFR) levels) were assessed in the 12-month baseline period and the post-index period (including the index date).

HCRU and healthcare costs were assessed during the 12-month post-index period, including the index date. HCRU was evaluated based on ambulatory visits (physician office and hospital outpatient visits reported for rheumatology, nephrology, primary care, and other specialties), emergency room visits, inpatient stays, and pharmacy use. Healthcare costs included medical and pharmacy costs and were adjusted using the annual medical care component of the 2019 USD Consumer Price Index to reflect inflation. Medical costs included ambulatory costs (physician office and hospital outpatient visits reported for rheumatology, nephrology, primary care, and other specialties), emergency room costs, inpatient costs, and other medical costs (costs of services rendered at independent laboratories, assisted living facilities, urgent care clinics, and by home health providers).

SLE clinical manifestations were identified using ICD-10 diagnosis codes, which were recorded for up to 12 months after the index date.

The number, length, and severity of SLE and LN flares experienced in the post-index period were reported based on the algorithm developed by Garris et al., which combines HCRU, ICD-10 diagnosis codes, and medication use to categorize flare severity (renal components were used for LN flares; as a renal diagnosis code constitutes a moderate severity flare, LN flares can only be of moderate or severe severity) [21]. LN-related events such as renal biopsy, chronic kidney disease (CKD), and ESKD (identified using ICD-9 and ICD-10 diagnosis codes) were also reported.

SLE- and LN-specific treatment use was assessed during the pre- and post-index periods using medical and pharmacy claims; claims made on the index date were counted as post-index.

Statistical analysis

All variables were analyzed descriptively. Numbers and percentages are provided for dichotomous and polychotomous variables. Means and standard deviation (SD) are reported for continuous variables.

Patients in the LN cohort were matched in a one-to-one ratio with patients in the SLE without LN cohort on age group, sex, geographic region, insurance type (commercial or Medicare Advantage), and index month and year. Matching also required patients to have a less than 10-year difference in age and a maximum of six-month difference in the index date. The closest match for each was used; ties were settled at random. Patients in the SLE without LN cohort who could not be matched to patients in the LN cohort were excluded from the analysis. A Z-test using robust standard errors in an ordinary least squares regression was used for continuous measures; a Rao-Scott test was used for binary measures.

## Results

Patient demographics and clinical characteristics

After matching, a total of 4620 patients were included in the analysis (LN: n=2310; SLE without LN: n=2310). Of these, 1088 patients were newly diagnosed (LN: n=564; SLE without LN: n=524).

Demographic characteristics were similar between the LN and SLE without LN cohorts. Patients had a mean (standard deviation (SD)) age of 60.2 (15.4) and 60.2 (15.3) years, respectively; 44.2% (each cohort: n=1020/2310) were ≥65 years of age, the majority were female (each cohort: 86.2% (n=1990/2310)), from the South region of the USA (each cohort: 58.1% (n=1341/2310)), and 66.3% (each cohort: n=1531/2310) had Medicare Advantage insurance (Table 1).

**Table 1:** Patient demographics and baseline clinical characteristics among LN and matched SLE without LN cohorts. ^a^AHRQ CCS comorbidities assigned from the ICD-10 coding scheme [23]. ^b^Comprised of anemia (LN cohort: 690 (29.9%), SLE without LN cohort: 325 (14.1%)), thrombophilia (LN cohort: 220 (9.5%), SLE without LN cohort: 153 (6.6%)), thrombocytopenia (LN cohort: 209 (9.1%), SLE without LN cohort: 110 (4.8%)), neutropenia/leukopenia (LN cohort: 177 (7.7%), SLE without LN cohort: 112 (4.9%)), lymphadenopathy/splenomegaly (LN cohort: 29 (1.3%), SLE without LN cohort: 26 (1.1%)), and lymph node enlargement (LN cohort: 125 (5.4%), SLE without LN cohort: 68 (2.9%)). ^c^Identified as the presence of an ICD-10 diagnosis code for ESKD or the onset of chronic renal dialysis indicated by a Current Procedural Terminology dialysis code or an ICD-10 dialysis diagnosis code. AHRQ: Agency for Healthcare Research and Quality; CCS: Clinical Classifications Software; CKD: chronic kidney disease; eGFR: estimated glomerular filtration rate; ESKD: end-stage kidney disease; ICD: International Classification of Diseases; LN: lupus nephritis; SD: standard deviation; SLE: systemic lupus erythematosus; uPCR: urinary protein/creatinine ratio.

Baseline characteristics	LN (N=2310)	SLE without LN (N=2310)	p-value
Demographics			
Age, years, mean (SD)	60.2 (15.4)	60.2 (15.3)	0.888
Age group, n (%)			
18–44	395 (17.1)	395 (17.1)	-
45–64	895 (38.7)	895 (38.7)	-
≥65	1020 (44.2)	1020 (44.2)	-
Female, n (%)	1990 (86.2)	1990 (86.2)	-
Region, n (%)			
Northeast	191 (8.3)	191 (8.3)	-
Midwest	516 (22.3)	516 (22.3)	-
South	1341 (58.1)	1341 (58.1)	-
West	262 (11.3)	262 (11.3)	-
Insurance type, n (%)			
Commercial	779 (33.7)	779 (33.7)	-
Medicare Advantage	1531 (66.3)	1531 (66.3)	-
Newly diagnosed	564 (24.4)	524 (22.7)	
Clinical characteristics			
Index provider specialty, n (%)			
Nephrologist	654 (28.3)	12 (0.5)	<0.001
Rheumatologist	285 (12.3)	848 (36.7)	<0.001
Prescribing provider specialty visits, mean (SD)			
Rheumatologist	2.3 (2.8)	2.2 (2.9)	0.238
Nephrologist	3.5 (8.6)	0.1 (0.4)	<0.001
Primary care physician	11.5 (14.7)	6.9 (7.4)	<0.001
Baseline Charlson comorbidity score, mean (SD) [22]	3.3 (2.1)	1.8 (1.5)	<0.001
Most common AHRQ CCS comorbidities^a^ (top six of the LN cohort), n (%)			
Other diseases of the urinary system	2012 (87.1)	956 (41.4)	<0.001
Hypertension	1973 (85.4)	1371 (59.4)	<0.001
Non-traumatic joint disorders	1574 (68.1)	1603 (69.4)	0.346
Other connective tissue diseases	1520 (65.8)	1471 (63.7)	0.124
Diseases of the heart	1514 (65.5)	1087 (47.1)	<0.001
Disorders of lipid metabolism	1403 (60.7)	1093 (47.3)	<0.001
Most common clinical manifestations (top six of the LN cohort), n (%)			
Arthralgia	1102 (47.7)	1126 (48.7)	0.475
Hematologic disorders^b^	1033 (44.7)	626 (27.1)	<0.001
Rheumatoid arthritis	536 (23.2)	604 (26.2)	0.020
Ophthalmologic disorders	492 (21.3)	478 (20.7)	0.604
Fever	342 (14.8)	173 (7.5)	<0.001
Rash	240 (10.4)	235 (10.2)	0.806
Laboratory tests			
Proteinuria concentration (mg/dL)			
Patients with proteinuria concentration test, n (%)	836 (36.2)	479 (20.7)	<0.001
Average per-patient counts of proteinuria concentration tests on separate dates, mean (SD)	2.5 (2.4)	1.7 (1.4)	<0.001
Average overall level of proteinuria concentration during the baseline period, mean (SD)	105.1 (326.9)	23.8 (177.3)	<0.001
uPCR (mg/g)			
Patients with uPCR, n (%)	590 (25.5)	224 (9.7)	<0.001
Average per-patient counts of uPCR on separate dates, mean (SD)	2.3 (2.1)	1.5 (1.3)	<0.001
Average overall uPCR during the baseline period, mean (SD)	737.8 (1355.6)	89.7 (177.6)	<0.001
eGFR (mL/min/1.73 m^2^)			
Patients with eGFR test, n (%)	1246 (53.9)	1157 (50.1)	0.007
Average per-patient counts of eGFR tests on separate dates, mean (SD)	3.7 (3.3)	2.5 (1.9)	<0.001
Average overall eGFR level during the baseline period, mean (SD)	58.6 (28.9)	84.1 (17.9)	<0.001
LN-related events, n (%)			
Renal biopsy	119 (5.2)	0 (0)	-
CKD			
None	354 (15.3)	1119 (48.4)	<0.001
Stage I	153 (6.6)	371 (16.1)	<0.001
Stage II	288 (12.5)	634 (27.5)	<0.001
Stage III	987 (42.7)	181 (7.8)	<0.001
Stage IV	268 (11.6)	3 (0.1)	<0.001
Stage V	260 (11.3)	2 (0.1)	<0.001
ESKD^c^	267 (11.6)	1 (0)	<0.001

The mean (SD) baseline Charlson comorbidity score was higher in the LN cohort compared with the SLE without the LN cohort (3.3 (2.1) vs 1.8 (1.5), respectively; p<0.001). Patients in the LN cohort were more likely to have comorbidities such as hypertension, other diseases of the urinary system, diseases of lipid metabolism, and diseases of the heart compared with patients in the SLE without LN cohort at baseline (p<0.001) (Table 1). Patients in the LN cohort also experienced more baseline hematologic disorders and fever compared with those in the SLE without LN cohort (Table 1).

Among patients for whom renal laboratory data were available, the average baseline proteinuria and uPCR levels for patients in the LN cohort were over four times and over eight times those of the patients in the SLE without LN cohort (p<0.001), respectively, and the eGFR rates of the SLE without LN cohort were higher than those of the LN cohort (p<0.001) (Table 1). A significantly higher proportion of patients in the LN cohort had stages III, IV, or V CKD and ESKD compared with those in the SLE without LN cohort at baseline (p<0.001) (Table 1).

HCRU

**Figure 2:**
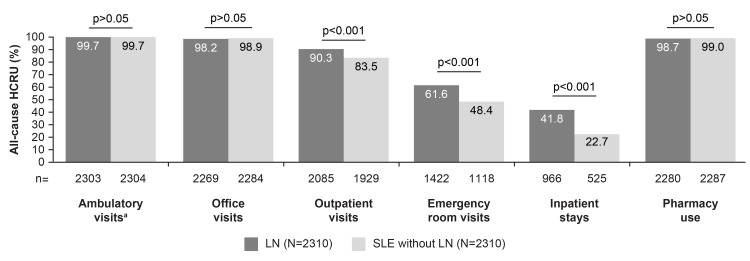
Percentages of patients with all-cause HCRU among LN and matched SLE without LN cohorts post-index. ^a^Ambulatory visits include all-cause physician office visits and/or hospital outpatient visits to rheumatology, nephrology, primary care, and other specialties. HCRU: healthcare resource utilization; LN: lupus nephritis; SLE: systemic lupus erythematosus.

**Figure 3:**
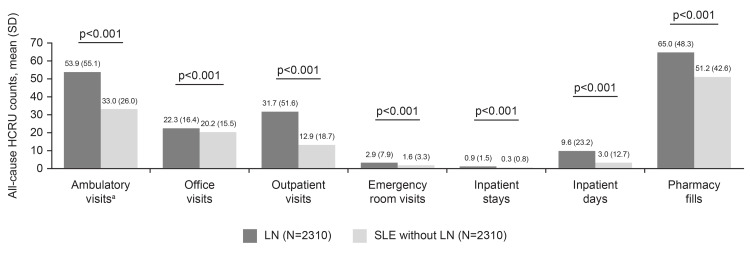
All-cause HCRU counts among LN and matched SLE without LN cohorts post-index. ^a^Ambulatory visits include all-cause physician office visits and/or hospital outpatient visits to rheumatology, nephrology, primary care, and other specialties. HCRU: healthcare resource utilization; LN: lupus nephritis; SD: standard deviation; SLE: systemic lupus erythematosus.

During the post-index period, a higher proportion of patients in the LN cohort experienced all-cause HCRU outpatient visits (90.3% (n=2085/2310) vs 83.5% (n=1929/2310), p<0.001), emergency room visits (61.6% (n=1422/2310) vs 48.4% (n=1118/2310), p<0.001), and inpatient stays (41.8% (n=966/2310) vs 22.7% (n=525/2310), p<0.001) compared with those in the SLE without LN cohort, whereas similar proportions of the two cohorts incurred all-cause HCRU physician office visits (98.2% (n=2269/2310) vs 98.9% (n=2284/2310)) and pharmacy use (98.7% (n=2280/2310) vs 99.0% (n=2287/2310); Figure 2). In all settings, the mean (SD) use of healthcare resources was higher in the LN cohort compared with the SLE without LN cohort, including the mean number of ambulatory visits (53.9 (55.1) vs 33.0 (26.0), p<0.001), emergency room visits (2.9 (7.9) vs 1.6 (3.3), p<0.001), inpatient stays (0.9 (1.5) vs 0.3 (0.8), p<0.001), and pharmacy fills (65.0 (48.3) vs 51.2 (42.6), p<0.001; Figure 3). Ambulatory visits were reported for rheumatology, nephrology, primary care, and other specialties.

Healthcare costs

**Figure 4:**
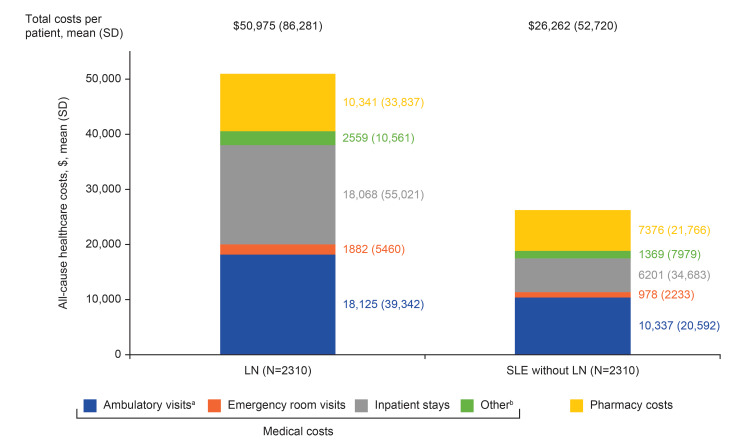
All-cause healthcare costs among LN and matched SLE without LN cohorts post-index. ^a^Ambulatory visits include all-cause physician office visits and/or hospital outpatient visits to rheumatology, nephrology, primary care, and other specialties. ^b^Other includes costs for services rendered at independent laboratories, assisted living facilities, urgent care clinics, and by home health providers. LN: lupus nephritis; SD: standard deviation; SLE: systemic lupus erythematosus.

In the post-index period, mean (SD) all-cause total costs per patient in the LN cohort were significantly higher compared with the SLE without LN cohort ($50,975 (86,281) vs $26,262 (52,720), p<0.001, Figure 4). Furthermore, each healthcare category showed higher mean (SD) costs in the LN cohort, with inpatient stay costs almost three times those in the SLE cohort ($18,068 (55,021) vs $6201 (34,683)). Within the ambulatory visit costs category, mean (SD) costs for physician office visits were not significantly different between cohorts (LN: $4116 (8396) vs SLE without LN: $4367 (10,882), p=0.382), but costs for outpatient visits were significantly higher in the LN cohort compared with the SLE without LN cohort ($14,009 (37,827) vs $5970 (16,766), p<0.001).

Clinical manifestations and renal laboratory measurements

A higher proportion of patients with LN experienced hematologic disorders and fever during the post-index period than patients with SLE without LN (49.5% (n=1143/2310) and 15.7% (n=363/2310), respectively, vs 28.8% (n=666/2310) and 8.8% (n=203/2310), respectively; p<0.001), whereas both groups had similar rates of arthralgia, ophthalmologic disorders, rash, Raynaud's phenomenon, and mouth ulcers (Table 2).

**Table 2:** Clinical manifestations among patients with LN and patients with SLE without LN post-index. LN: lupus nephritis; SLE: systemic lupus erythematosus.

Clinical manifestations, n (%)	LN (N=2310)	SLE without LN (N=2310)	p-value
Hematologic disorders	1143 (49.5)	666 (28.8)	<0.001
Anemia	788 (34.1)	369 (16.0)	<0.001
Thrombophilia	244 (10.6)	143 (6.2)	<0.001
Thrombocytopenia	246 (10.7)	119 (5.2)	<0.001
Neutropenia/leukopenia	188 (8.1)	109 (4.7)	<0.001
Lymph node enlargement	117 (5.1)	79 (3.4)	0.004
Lymphadenopathy/splenomegaly	44 (1.9)	21 (0.9)	0.004
Arthralgia	1103 (47.8)	1240 (53.7)	<0.001
Ophthalmologic disorders	523 (22.6)	558 (24.2)	0.219
Fever	363 (15.7)	203 (8.8)	<0.001
Rash	222 (9.6)	245 (10.6)	0.256
Raynaud's phenomenon	175 (7.6)	190 (8.2)	0.422
Mouth ulcers	36 (1.6)	42 (1.8)	0.486

Over the post-index period, among patients for whom renal laboratory data were available, the average proteinuria and uPCR levels for patients in the LN cohort were almost five times and over five times those of the patients in the SLE without LN cohort (p<0.001), respectively, and the eGFR rates of the SLE without LN cohort were higher than those of the LN cohort (p<0.001) (Table 3).

**Table 3:** Renal laboratory tests post-index. eGFR: estimated glomerular filtration rate; LN: lupus nephritis; SD: standard deviation; uPCR: urinary protein/creatinine ratio.

Laboratory tests	LN (N=2310)	SLE without LN (N=2310)	p-value
Proteinuria concentration (mg/dL)			
Patients with proteinuria concentration test, n (%)	866 (37.5)	540 (23.4)	<0.001
Average per-patient counts of proteinuria concentration tests on separate dates, mean (SD)	2.8 (3.3)	1.8 (1.4)	<0.001
Average overall level of proteinuria concentration during the post-index period, mean (SD)	94.9 (274.1)	19.3 (113.4)	<0.001
uPCR (mg/g)			
Patients with uPCR, n (%)	637 (27.6)	272 (11.8)	<0.001
Average per-patient counts of uPCR on separate dates, mean (SD)	2.5 (2.3)	1.7 (1.4)	<0.001
Average overall uPCR during the post-index period, mean (SD)	634.7 (1176.8)	120.2 (322.4)	<0.001
eGFR (mL/min/1.73 m^2^)			
Patients with eGFR test, n (%)	1228 (53.2)	1240 (53.7)	0.711
Average per-patient counts of eGFR tests on separate dates, mean (SD)	3.8 (3.8)	2.7 (2.2)	<0.001
Average overall eGFR level during the post-index period, mean (SD)	57.1 (28.7)	82.3 (18.3)	<0.001

Flares

Most patients in both the LN and SLE without LN cohorts had ≥1 SLE flare of any severity during the post-index period (98.8% (n=2282/2310) and 95.5% (n=2205/2310), respectively; Table 4). A significantly higher proportion of patients with LN experienced moderate (95.7% (n=2210/2310) vs 86.7% (n=2002/2310)) and severe SLE flares (31.7% (n=731/2310) vs 12.5% (n=288/2310)) compared with patients with SLE without LN (all p<0.001).

**Table 4:** Counts and severity of flares among patients with LN and patients with SLE without LN post-index. ^a^Individual patients may have ≥1 flare. LN: lupus nephritis; SD: standard deviation; SLE: systemic lupus erythematosus.

SLE flares	LN (N=2310)	SLE without LN (N=2310)	p-value
SLE flare^a^			
Patients with any SLE flare, n (%)	2282 (98.8)	2205 (95.5)	<0.001
Total SLE flare count, mean (SD)	5 (2.2)	4 (2.2)	<0.001
Patients with mild flare, n (%)	1194 (51.7)	1435 (62.1)	<0.001
Mild flare count, mean (SD)	1.0 (1.3)	1.4 (1.4)	<0.001
Patients with moderate flare, n (%)	2210 (95.7)	2002 (86.7)	<0.001
Moderate flare count, mean (SD)	3.5 (1.7)	2.5 (1.7)	<0.001
Patients with severe flare, n (%)	731 (31.7)	288 (12.5)	<0.001
Severe flare count, mean (SD)	0.5 (0.9)	0.2 (0.4)	<0.001
LN flares^a^			
Patients with any LN flare, n (%)	2044 (88.5)	66 (2.9)	<0.001
Total LN flare count, mean (SD)	2.3 (1.5)	0 (0.2)	<0.001
Patients with moderate flare, n (%)	1925 (83.3)	60 (2.6)	<0.001
Moderate flare count, mean (SD)	2.0 (1.4)	0 (0.2)	<0.001
Patients with severe flare, n (%)	447 (19.4)	7 (0.3)	<0.001
Severe flare count, mean (SD)	0.3 (0.7)	0 (0.1)	<0.001

Whereas in the LN cohort, most patients had ≥1 LN flare of any severity post-index (88.5% (n=2044/2310)), this was not the case in the SLE without LN cohort (2.9% (n=66/2310), p<0.001, Table 4). Additionally, a significantly higher proportion of patients with LN experienced moderate (83.3% (n=1925/2310) vs 2.6% (n=60/2310)) and severe LN flares (19.4% (n=447/2310) vs 0.3% (n=7/2310)) compared with patients with SLE without LN (all p<0.001).

Treatments

The most frequently used treatments among patients with LN included corticosteroids (70.1% (n=1619/2310)), angiotensin-converting enzyme inhibitors (ACEis)/angiotensin receptor blockers (ARBs; 52.0% (n=1200/2310)), and antimalarials (51.3% (n=1184/2310)), whereas the most frequently used treatments among patients with SLE without LN were corticosteroids (69.1% (n=1597/2310)), antimalarials (58.3% (n=1346/2310)), and nonsteroidal anti-inflammatory drugs (NSAIDs; 37.5% (n=866/2310); Table 5). Significantly more patients with LN received oral corticosteroids, ACEis/ARBs, and immunosuppressants compared with patients with SLE without LN (p<0.001). In contrast, more patients with SLE without LN received IV corticosteroids, antimalarials, and NSAIDs compared with patients with LN (p<0.001).

**Table 5:** Treatments post-index. ACEis/ARBs: angiotensin-converting enzyme inhibitors/angiotensin receptor blockers; IV: intravenous; LN: lupus nephritis; NSAIDs: nonsteroidal anti-inflammatory drugs; SLE: systemic lupus erythematosus.

SLE and LN specific treatments, n (%)	LN (N=2310)	SLE without LN (N=2310)	p-value
Corticosteroids	1619 (70.1)	1597 (69.1)	0.477
IV	888 (38.4)	1001 (43.3)	<0.001
Oral	1363 (59.0)	1223 (52.9)	<0.001
Patients ≥5 mg prednisone equivalent per day (dose/day supply)			
Post-index months 1–6	1066 (78.2)	932 (76.2)	0.226
Post-index months 7–12	947 (69.5)	816 (66.7)	0.135
Patients ≥7.5 mg prednisone equivalent per day (dose/day supply)			
Post-index months 1–6	806 (59.1)	766 (62.6)	0.070
Post-index months 7–12	693 (50.8)	662 (54.1)	0.097
ACEis/ARBs	1200 (52.0)	678 (29.4)	<0.001
Antimalarials	1184 (51.3)	1346 (58.3)	<0.001
Immunosuppressants	760 (32.9)	532 (23.0)	<0.001
Methotrexate	120 (5.2)	262 (11.3)	<0.001
Mycophenolate	490 (21.2)	136 (5.9)	<0.001
Cyclophosphamide	31 (1.3)	5 (0.2)	<0.001
Azathioprine	178 (7.7)	165 (7.1)	0.460
NSAIDs	477 (20.7)	866 (37.5)	<0.001
Immunosuppressant biologics	112 (4.9)	135 (5.8)	0.134
Rituximab	52 (2.3)	32 (1.4)	0.029
Benlysta	62 (2.7)	105 (4.6)	<0.001

## Discussion

This retrospective observational study illustrated the increased economic and clinical burden associated with LN, as represented by the greater HCRU, costs of care, and frequency of flares and comorbidities for patients with LN versus matched patients with SLE without LN. These results highlight the importance of preventing SLE disease worsening and preventing the development of renal manifestations. The greatest difference in HCRU was driven by outpatient visits, emergency room visits, and inpatient stays. Total costs, which included medical and pharmacy costs, were almost double for patients with LN compared with patients with SLE without LN. In particular, the cost of inpatient stays was approximately three times higher for the LN cohort compared with the SLE without LN cohort.

The economic findings are consistent with a previous study by Pelletier et al., which found that patients with LN incurred substantially greater HCRU and higher healthcare costs compared with patients with SLE without LN, driven primarily by inpatient admissions and outpatient visits [15]. Pelletier et al. also reported that the mean total costs for patients with LN were 89% higher than for those with SLE without LN (US$21,733 vs US$11,471; 2008 values) [15]. Similarly, Carls et al. found that the mean total direct medical costs for patients with SLE without LN were significantly higher compared with matched controls without SLE (US$15,447 vs US$6819; difference: US$8628; 2005 values) [16]. However, the difference was much higher when comparing patients with LN with matched controls without SLE (US$58,389 vs US$11,527; difference: US$46,862; 2005 values) [16]. Li et al. also reported that annual medical costs for patients with LN were approximately two to three times those of patients with SLE without LN, and the number of inpatient visits were three to four times those of the control cohort without SLE [18].

Regarding the clinical burden of LN, more patients with LN had hematologic disorders and fever than patients with SLE without LN, and proteinuria and uPCR levels were almost five times and over five times those of the patients with SLE without LN, respectively. Patients with LN were also more likely to experience moderate or severe SLE or LN flares than patients with SLE without LN, and significantly more patients with LN received oral corticosteroids. The increased risk of flares could help to explain why patients with LN have higher HCRU and healthcare costs compared with patients with SLE without LN, as the occurrence of SLE flares has previously been associated with higher HCRU and costs when compared with patients without flares [24]. Future analyses could further evaluate the costs of flares and LN progression by stratifying the HCRU and healthcare costs of patients with LN, with and without ESKD or dialysis. Patients with LN also had lower average eGFR rates compared with patients with SLE without LN. Given that renal flares are commonly associated with impaired renal function and CKD [7-9], and a single renal flare is sufficient to cause kidney damage that can affect the lifespan of the kidney [10], preventing renal flares is an important strategy for managing disease burden in patients with LN.

Finally, the most frequently used post-index treatments among both cohorts were corticosteroids and antimalarials; however, ACEis and ARBs were also frequently used in patients with LN, while NSAIDs were used in patients with SLE without LN. Corticosteroids have been associated with short- and long-term adverse events (including high blood pressure and glucose levels, sleep disorders, weight gain, cataracts, osteoporosis, and diabetes) and organ damage in SLE [25-27]. Therefore, the frequent use of corticosteroids (including doses ≥7.5 mg of prednisone equivalent per day) in both cohorts may highlight an unmet need for corticosteroid-sparing treatments for SLE and LN. Interestingly, while overall corticosteroid use was similar between the two groups, corticosteroid use differed considerably based on formulation; more patients with LN used oral corticosteroids versus patients with SLE without LN, while conversely, fewer patients with LN used intravenous corticosteroids compared with patients with SLE without LN. A previous study by Pelletier et al. demonstrated that patients with LN were more likely to be treated with immunosuppressants or corticosteroids compared with patients with SLE without LN [15]. Rates of nonbiologic immunosuppressant use in the current study were also higher among patients with LN compared with those with SLE without LN, although immunosuppressants were not used as frequently as other treatment classes. Both studies showed higher rates of corticosteroid use in patients with LN compared with those with SLE without LN. The higher use of ACEis and ARBs we observed is likely related to the increased rates of hypertension and proteinuria in our LN cohort versus the SLE without LN cohort. The higher use of NSAIDs in patients with SLE without LN was expected as, while it is a common treatment in SLE, this is a contraindication for patients with CKD [28].

This study has several limitations, most of which are inherent to observational studies based on claims data. These include the potential for data coding and entry errors, the possibility of incorrect diagnoses, the inability to confirm that a patient took the medication as prescribed, and the fact that the claims databases do not capture indirect costs. Additionally, the study population was limited to patients with commercial or Medicare Advantage insurance coverage, so the results may not be generalizable to the general population. In particular, the inclusion of Medicare Advantage patients may have skewed the population older than is typical for LN [4,18,29]. Further limitations include the use of renal manifestation diagnosis codes to identify LN, rather than using kidney biopsy results, and the algorithm used in this study for identifying flares may not accurately capture renal flares among patients with LN. We used a previously validated claims algorithm to identify patients and assigned them to the cohort of patients with LN, when in fact these patients may not have been formally diagnosed with LN [20]. In addition, adopting definitions of renal flares based on laboratory assessments, as in the phase III BLISS-LN trial assessing the effects of belimumab on kidney outcomes in patients with LN [30], may improve the accuracy of flare reporting in future studies. A further limitation is that laboratory results were only available for a subset of patients and may have been incomplete. Finally, patients who were excluded due to the matching procedure may represent a specific population, which could introduce bias.

Nevertheless, this study had several strengths as well, including using a data source that represented a broad and geographically diverse sample of insured patients in a real-world setting across the USA, and a matched control cohort that was used to control for potential confounding differences among patient groups at baseline.

## Conclusions

This analysis of real-world data from the USA demonstrates that all-cause HCRU and healthcare costs were significantly higher for patients with LN than those with SLE without LN. In particular, patients with LN experienced a higher mean number of ambulatory visits, emergency room visits, inpatient stays, and pharmacy fills compared with patients with SLE without LN. This highlights the substantial additional economic burden associated with LN. The results also demonstrate the increased clinical burden for patients with LN, underscoring the importance of preventing SLE disease worsening and preventing the development of renal manifestations. In the future, it would be of interest to investigate how renal involvement affects the HCRU and healthcare costs of patients with other systemic diseases compared with LN.
